# Effectiveness of Whey Protein Supplementation during Resistance Exercise Training on Skeletal Muscle Mass and Strength in Older People with Sarcopenia: A Systematic Review and Meta-Analysis

**DOI:** 10.3390/nu15153424

**Published:** 2023-08-02

**Authors:** Iván Cuyul-Vásquez, José Pezo-Navarrete, Cristina Vargas-Arriagada, Cynthia Ortega-Díaz, Walter Sepúlveda-Loyola, Sandro Massao Hirabara, Gabriel Nasri Marzuca-Nassr

**Affiliations:** 1 Departamento de Procesos Terapéuticos, Facultad de Ciencias de la Salud, Universidad Católica de Temuco, Temuco 4813302, Chile; icuyul@uct.cl (I.C.-V.); jpezo2017@alu.uct.cl (J.P.-N.); cristina.vargas2018@alu.uct.cl (C.V.-A.); cynthia.ortega2018@alu.uct.cl (C.O.-D.); 2 Facultad de Ciencias de la Salud, Universidad Autónoma de Chile, Temuco 4810101, Chile; 3 Faculty of Health and Social Sciences, Universidad de Las Americas, Santiago 8370040, Chile; wsepulveda@udla.cl; 4 Interdisciplinary Post-graduate Program in Health Sciences, Cruzeiro do Sul University, São Paulo 01506-000, Brazil; sandro.hirabara@cruzeirodosul.edu.br; 5 Departamento de Ciencias de la Rehabilitación, Facultad de Medicina, Universidad de La Frontera, Claro Solar 115, Temuco 4811230, Chile; 6 Interuniversity Center for Healthy Aging, Talca 3460000, Chile

**Keywords:** Sarcopenia, elderly, whey protein, resistance exercise, strength training

## Abstract

Objective: To determine the effectiveness of whey protein (WP) supplementation during resistance exercise training (RET) vs. RET with or without placebo supplementation on skeletal muscle mass, strength, and physical performance in older people with Sarcopenia. Methods: Electronic searches in the PubMed, Embase, Scopus, Web of Science, LILACS, SPORTDiscus, Epistemonikos, and CINAHL databases were performed until 20 January 2023. Randomized clinical trials conducted on sarcopenic adults aged 60 or older were included. The studies had to compare the effectiveness of the addition of supplements based on concentrated, isolated, or hydrolyzed whey protein during RET and compare it with RET with or without placebo supplementation on skeletal muscle mass and strength changes. The study selection process, data extraction, and risk of bias assessment were carried out by two independent reviewers. Results: Seven randomized clinical trials (591 participants) were included, and five of them provided data for quantitative synthesis. The overall pooled standardized mean difference (SMD) estimate showed a small effect size in favor of RET plus WP for skeletal muscle mass according to appendicular muscle index, with statistically significant differences compared with RET with or without the placebo group (SMD = 0.24; 95% CI, 0.05 to 0.42; *p* = 0.01; *I*^2^ = 0%, *p* = 0.42). The overall pooled mean difference (MD) estimate showed a significant difference of +2.31 kg (MD = 2.31 kg; 95% CI, 0.01 to 4.6; *p* = 0.05; *I*^2^ = 81%, *p* < 0.001) in handgrip strength in the RET plus WP group compared with the RET group with or without placebo. The narrative synthesis revealed discordance between the results of the studies on physical performance. Conclusions: WP supplementation during RET is more effective in increasing handgrip strength and skeletal muscle mass in older people with Sarcopenia compared with RET with or without placebo supplementation. However, the effect sizes were small, and the MD did not exceed the minimally important clinical difference. The quality of the evidence was low to very low according, to the GRADE approach. Further research is needed in this field.

## 1. Background

Sarcopenia is a condition characterized by decreased skeletal muscle mass, muscle strength, and physical performance [1]. Sarcopenia has been positively correlated with elevated rates of disability, hospitalization, falls, fractures, and mortality risk in older adults [2,3]. Compared with older adults without Sarcopenia, the deterioration of physical performance is 3 times higher, and the mortality rate is 3.6 times higher in older adults with Sarcopenia [3]. Individuals with Sarcopenia present an increased risk of hospitalization for different adverse events [4]. For these reasons, preventing and treating Sarcopenia is essential to reducing healthcare costs [4]. In this sense, different interventions have been recommended, including exercise training and nutritional supplementation [5]. Progressive resistance exercise training (RET) of moderate to high intensity is one of the interventions with the highest degree of recommendation for the prevention and treatment of Sarcopenia [6,7,8].

RET has been shown to increase skeletal muscle mass, muscle strength, and physical performance in healthy older people or those with an increased risk of Sarcopenia [9,10,11,12]. Furthermore, RET has been shown to be superior in improving muscle strength of the upper and lower extremities, handgrip strength, depressive symptoms, physical performance, walking speed, and distance compared with other exercise modalities in healthy, sarcopenic, and hospitalized older people [9,10,11,12,13]. In addition, RET is an excellent, cost-effective modality for reducing frailty and the risk of falls in healthy older people [14,15]. Similar results have been observed in older people with Sarcopenia on muscle strength and physical performance; however, the effects of RET on skeletal muscle mass are heterogeneous [16,17,18]. According to a recent meta-analysis, the effect of RET on skeletal muscle mass is still controversial since RET showed a small effect size (SMD = 0.28) on lower limb skeletal muscle mass but not overall or on upper limb skeletal muscle mass compared with education or maintaining the daily lifestyle in people with Sarcopenia [16].

Another important recommendation to treat and/or prevent Sarcopenia is protein supplementation [5]. As in healthy older people [19,20], it has been suggested that oral Whey Protein (WP) could maximize the effects of exercise and positively influence skeletal muscle anabolism in people with Sarcopenia [21]. In older people, protein supplementation has been shown to increase overall lean mass and handgrip strength only when combined with RET [22]. For example, WP plus leucine intake has been shown to induce postprandial increases in plasma amino acid levels and stimulate muscle protein synthesis in people with Sarcopenia to a greater extent than any other protein source [23]. Regarding the use of WP, a daily dose of 20–40 g combined with RET has been shown to increase biceps strength and lower limb lean mass in post-menopausal women [24].

Although there is plausibility for the potential benefit of the combination of WP with RET in older people with Sarcopenia, the magnitude of the effects on skeletal muscle mass, muscle strength, and physical performance is still unknown. Therefore, the aim of this systematic review was to determine the effectiveness of WP supplementation during RET vs. RET with or without placebo supplementation on skeletal muscle mass, muscle strength, and physical performance in older people with Sarcopenia.

## 2. Methods

### 2.1. Protocol and Registration

The report of this research was carried out according to the Preferred Reporting Items for Systematic Reviews and Meta-analysis (PRISMA) and the recommendations of the Cochrane Manual of Systematic Reviews of Interventions [25,26]. The protocol of this systematic review was published in PROSPERO with the registration number CRD42023391714.

### 2.2. Eligibility Criteria

Studies that met the following inclusion criteria were eligible: (1) population: adults 60 years of age or older, diagnosed with Sarcopenia (low skeletal muscle mass, muscle strength, and/or physical performance, according to the criteria of the international consensus of EWGSOP [1] or AWGSOP [27]), with or without concomitant diseases; (2) intervention: addition of supplements based on concentrated, isolated, or hydrolyzed WP during RET; RET was considered when the program training used machines, elastic bands, or free weights with moderate to high intensity (equal to or greater than 60% of 1 repetition maximum (1RM), for a minimum of 6 weeks); (3) comparison: moderate and high-intensity RET with or without placebo supplementation; (4) primary outcomes: skeletal muscle mass was measured using dual computed tomography scan, nuclear magnetic resonance, dual energy X-ray absorptiometry (DEXA), bioimpedance, or anthropometry; upper- and lower-limb muscle and grip strength were measured using dynamometry, 1RM, or load cell; physical performance was considered a secondary outcome: measurements conducted with the Short Physical Performance Battery (SPPB), Timed Up and Go (TUG), and walking speed were considered physical performance; and (5) types of studies: controlled clinical trials or randomized clinical trials published in English, Spanish, or Portuguese.

Studies were excluded if: (1) They were conducted in people with presarcopenia or dynapenia; (2) They were carried out with mixed samples of people with and without Sarcopenia; or (3) They were published only in conference proceedings.

### 2.3. Information Sources

Electronic searches were performed in Pubmed, Scopus, Web of Science (WOS), Embase, LILACS, SPORTDiscus, CINAHL, and Epistemonikos from the beginning of each database until January 2023. In addition, manual searches were performed on the references of the articles included in the electronic searches.

### 2.4. Search Strategies

Two independent reviewers (JP-N and CO-D) performed the electronic search in the databases. The search strategy was composed of the following MESH and free terms: “Sarcopenia”, “Sarcopenic”, “Sarcopaenia”, “Sarcopen”, “Strength training”, “Strength exercise”, “Weightlifting”, “Resistance training”, “Resistance exercise”, “Whey protein”, “Protein supplement”, “Whey supplement”, “Whey intake”, “Protein intake”, “Controlled Clinical Trial”, “Randomized controlled trial”, and “Clinical trial”. The search strategies for each database can be reviewed in Table S1 (Supplementary Materials).

### 2.5. Study Selection

The study selection process was carried out through the Rayyan collaborative web application [28]. Duplicates were eliminated before starting the article selection process. Subsequently, two independent reviewers (JP-N and CO-D) reviewed the titles and abstracts of the studies. Studies that did not meet the eligibility criteria were discarded. Potentially eligible studies were reviewed for full text. The agreement rate between reviewers for the study selection process was calculated using the Kappa statistic. Discrepancies between the reviewers’ assessments were discussed with a third reviewer (CV-A).

### 2.6. Data Collection Process

Data extraction was performed independently by two reviewers (JP-N and I-CV). The following information was extracted: Characteristics of the population (sample size, age, health status, and level of physical activity); intervention (type of intervention, supplements, dosage); and results (skeletal muscle mass, muscle strength, walking speed, physical performance, and dynamic balance).

### 2.7. Risk of Bias

Two independent reviewers (JP-N and WS-L) assessed studies using the Cochrane Risk of Bias 2 (RoB 2) tool [29]. ROB 2 has six domains: bias arising from the randomization process; bias due to deviations from intended interventions; bias due to missing outcome data; bias in measurement of the outcome; bias in selection of the reported result; and overall bias. Each domain could be considered “low risk”, “some concerns”, or “high risk” [29]. Discrepancies between reviewers’ assessments were discussed with a third reviewer (IC-V).

### 2.8. Statistical Methods

A quantitative synthesis was performed if there were at least three studies with comparable data. There were insufficient data to perform a meta-analysis of upper and lower limb strength (1RM), walking speed, SPPB, and TUG. A narrative synthesis of the effects of the interventions on physical performance was performed according to walking speed, SPPB, and TUG. A quantitative synthesis was performed for the skeletal muscle mass and handgrip strength outcomes. Mean differences (MD) or standardized mean differences (SMD) were calculated for each group. The calculation of the effect sizes considered the use of the raw baseline SD. A pooled estimate of the MD with 95% confidence intervals was calculated for handgrip strength (kg). A pooled estimate of the SMD with 95% confidence intervals was calculated for the appendicular muscle index and handgrip. The weighted sample size method was used to summarize effect sizes from multiple independent studies. Fixed-effects models with Mantel–Haenszel method or random-effects models with the DerSimonian-Laird method were used depending on the degree of heterogeneity. The *I*^2^ statistic was used to assess heterogeneity. For skeletal muscle mass, the effect size was considered trivial (SMD < 0.2), small (SMD 0.2–0.5), medium (SMD 0.6–0.8), or large (SMD > 0.8) [30]. Due to the lack of data for people with Sarcopenia, the threshold of 6.5 kg was considered a minimally important clinical difference for handgrip strength [31]. Subgroups were analyzed according to intervention time. Statistical significance was considered with a *p* value < 0.05. In the case of missing data, the reviewers contacted the authors by email. Meta-analysis would be performed using RevMan Manager 5.4 (Copenhagen, The Nordic Cochrane Centre, The Cochrane Collaboration).

### 2.9. Grading of Recommendation, Assessment, Development, and Evaluation

The synthesis and quality of evidence for skeletal muscle mass and muscle strength were assessed using the Grading of Recommendation, Assessment, Development, and Evaluation (GRADE) [32]. GRADE profile allows one to categorize the evidence as high, moderate, low, or very low quality [33]. Results of the GRADE analysis are shown in Table S3.

## 3. Results

### 3.1. Study Selection

**Figure 1:**
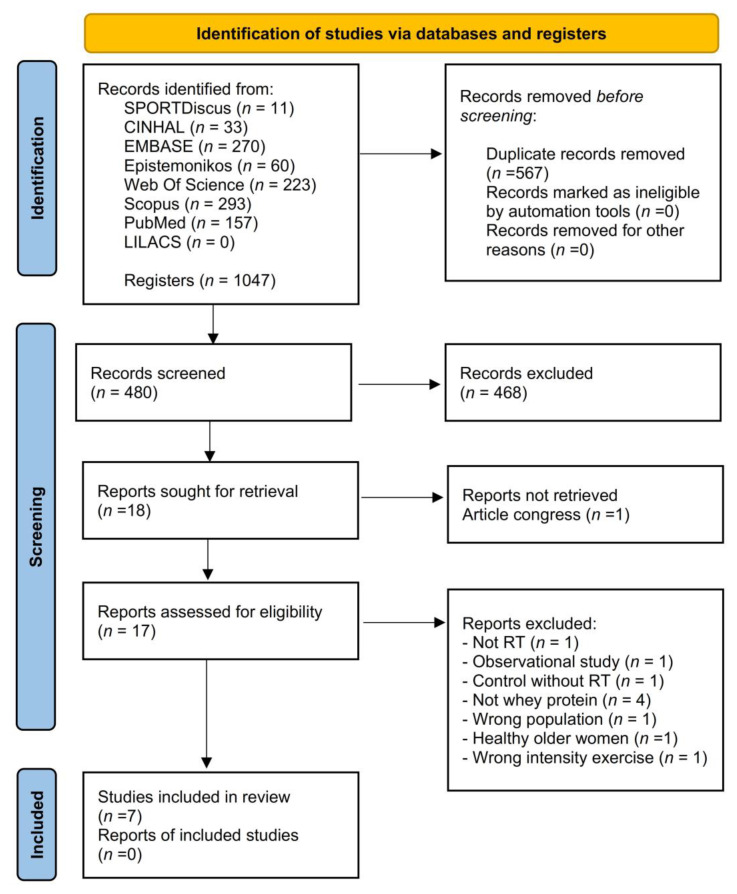
PRISMA flow diagram for the systematic review and meta-analysis.

A total of 1047 articles were found through electronic searches. Before starting the screening, 567 duplicate articles were eliminated, and a total of 480 articles were reviewed by title and abstract. Subsequently, 18 articles were reviewed in full text. The causes for the exclusion of articles can be seen in Table S2 (Supplementary Materials). Finally, seven randomized clinical trials were included [34,35,36,37,38,39,40]. The agreement rate between reviewers reached a kappa value of 0.91. Details of the study selection process are shown in Figure 1.

### 3.2. Study Characteristics

Table 1 summarizes the characteristics of the studies. The general population consisted of 591 people, 399 (67.5%) women, and 192 men (32.5%). The average age of the population was 77.3 years. All studies included untrained older adults. Four studies reported the inclusion of patients with comorbidities but did not report the details [34,36,37,40]. Three studies reported the inclusion of patients with obesity, hypertension, diabetes mellitus, dyslipidemia, osteoarthritis, chronic obstructive pulmonary disease, stroke, fractures, or a history of surgery [35,38,39]. Six studies were conducted in a hospital [34,35,36,37,38,40], or university outpatient setting [39]. Five studies included outpatients [34,35,38,39,40] and two included a mixed sample of inpatients and outpatients [36,37].

The duration of the intervention in the studies was 8 weeks [35], 12 weeks [34,36,37,38,39], or 24 weeks [40]. The studies used isolated [36,37] or hydrolyzed WP [39], and four studies did not report the type of WP [34,35,38,40]. Supplementation doses were 10–35 g. One study reported only the total protein count but did not detail the grams or percentage of WP [40]. Daily servings of WP ranged from 1–3 times per day. One study added no other ingredients to WP supplementation, while others added leucine [34,35,36,37,40], mineral vitamins [34,35,38], and/or essential polyunsaturated fatty acids [38]. Regarding the control groups, four studies added maltodextrin formulas supplementation to RET [34,35,36,37,39], and two studies used only RET [38,40]. The WP supplementation was performed in three studies only on training days [36,37,39]. In four studies, WP supplementation was administered every day during or after meals [34,35,38,40].

Regarding RET, four studies performed progressive RET until reaching 70% of 1RM [36,37,39,40]. One study used conventional RET with an intensity of 80% of 1RM [38]. Two studies used a conventional RET with moderate intensity (Borg 12–14) [34,35]. The frequency of RET sessions varied from 2–5 days per week. The duration of the RET sessions ranged from 20 to 60 min. Studies used machine, free weight, body weight, or elastic band training.

Five studies assessed skeletal muscle mass via DEXA [33,34,35,36] or multi-frecuency bioelectrical impedance analysis [38,40]. Muscle strength was evaluated in seven studies. The studies used handgrip dynamometry [34,35,36,37,38,40], 1RM [39], and maximum voluntary isometric contraction [40]. Five studies evaluated physical performance through different tests: SPPB [35,36], TUG [35], 10-m walk test [39], walk speed with 4 m test [35], and usual walking speed [40]. Regarding the confounding variables, four studies [34,35,39,40] and one study [40] controlled the diet intake and the physical activity during the execution of the interventions, respectively.

### 3.3. Risk of Bias Assessment

**Figure 2:**
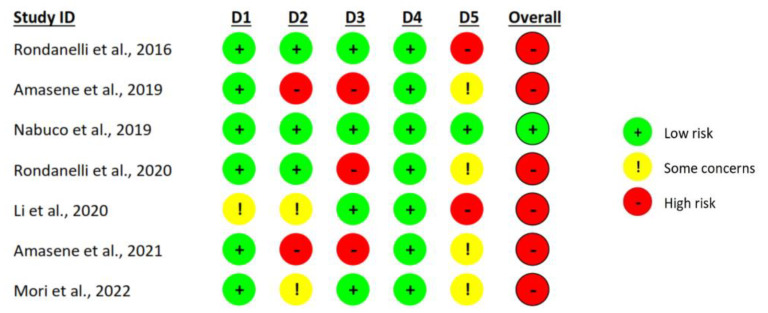
Risk of bias summary [34,35,36,37,38,39,40]. D1: Randomization process; D2: Deviations from the intended interventions; D3: Missing outcome data; D4: Measurement of the outcome; D5: Selection of the reported result.

**Figure 3:**
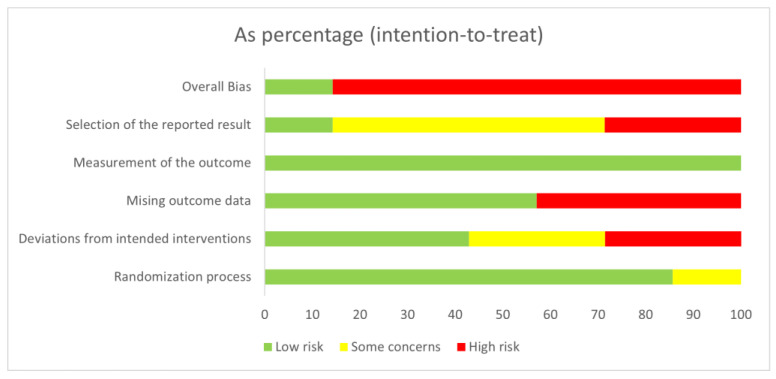
Risk of bias graph.

Figure 2 and Figure 3 show the results of the risk of bias assessment. As far as “overall bias” is concerned, 14.3% of the studies had a “low risk” and 85.7% had a “high risk”. Regarding the randomization process, 85.7% showed low risk, and 14.3% had some concerns. In the item deviations from intended interventions, 42.9% of the studies showed a low risk, 28.6% some concerns, and 28.6% a high risk. The studies showed a low risk of bias in 57.1% and a high risk of bias in 42.9% of the items missing outcome data. All studies showed a low risk of bias in the measurement of the outcome. In relation to the selection of the reported result, 14.3% of the studies showed a low risk, 57.1% some concerns, and 28.6% a high risk.

### 3.4. Synthesis of Results

#### 3.4.1. Skeletal Muscle Mass: Appendicular Muscle Index

**Figure 4:**
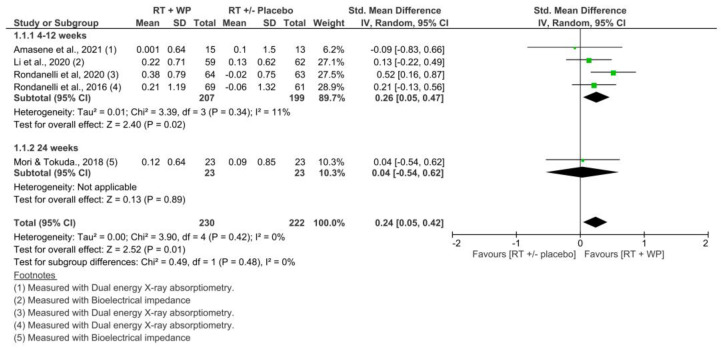
Comparison of RET plus WP vs. RET with or without placebo for appendicular muscle index, at 4 to 24 weeks and 4 to 12 weeks [34,35,36,38,40].

**Figure 5:**
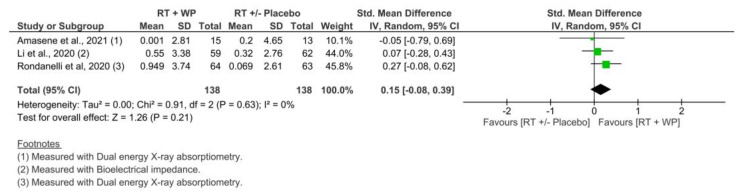
Comparison of RET plus WP vs. RET with or without placebo for appendicular muscle mass at 4 to 12 weeks [34,36,38].

Five studies included data on appendicular muscle index to perform the meta-analysis [34,35,36,38,40]. The overall pooled SMD estimate showed a small effect size in favor of RET plus WP supplementation with statistically significant differences compared with RET with or without the placebo group at 4–24 weeks (SMD = 0.24; 95% CI, 0.05 to 0.42; *p* = 0.01). No important heterogeneity was observed (*I*^2^ = 0%, *p* = 0.42) (Figure 4). There was a low quality of evidence, according to the GRADE rating. It was observed that weekly SMD varied between -0.01 and 0.13 in favor of the RET plus WP group (Table S4, Supplementary Materials). Four studies [34,35,36,38] with interventions lasting 4–12 showed a small effect size in favor of RET plus WP; however, there were no statistically significant differences compared with RET with or without a placebo group (SMD = 0.26; 95% CI, 0.05 to 0.47; *p* = 0.02), with no important heterogeneity (*I*^2^ = 11%, *p* = 0.34) (Figure 5). There was a low quality of evidence, according to the GRADE rating.

#### 3.4.2. Skeletal Muscle Mass: Appendicular Muscle Mass

Three studies [34,36,38] that included data on appendicular muscle mass to perform the meta-analysis showed a small effect size in favor of RET plus WP, however, without statistically significant differences compared with RET with or without the placebo group at 4–12 weeks (SMD = 0.15; 95% CI, −0.08 to 0.39; *p* = 0.21). Additionally, no important heterogeneity was observed (*I*^2^ = 0%, *p* = 0.63) (Figure 5). There was a low quality of evidence, according to the GRADE rating.

#### 3.4.3. Muscle Strength

**Figure 6:**
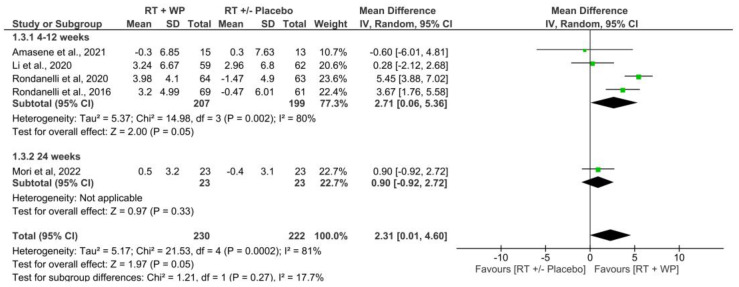
Comparison of RET plus WP vs. RET with or without placebo for handgrip strength at 4 to 24 weeks and 4 to 12 weeks [34,35,36,38,40].

Five studies included data on handgrip strength to perform the meta-analysis [34,35,36,38,40]. The overall pooled MD estimate showed a difference of +2.31 kg in handgrip strength in the RET plus WP supplementation compared with the RET group with or without placebo supplementation at 4–24 weeks, with statistically significant differences (MD = 2.31 kg; 95% CI, 0.01 to 4.6; *p* = 0.05) and considerable heterogeneity (*I*^2^ = 81%, *p* < 0.001) (Figure 6). There was a very low quality of evidence, according to the GRADE rating. Four studies [34,35,36,38] with interventions lasting 4–12 weeks showed a difference of +2.71 kg in handgrip strength in the RET plus WP group compared with the RET group with or without placebo supplementation at 4–24 weeks, with statistically significant differences (MD = 2.71 kg; 95% CI, 0.06 to 5.36; *p* = 0.05) and considerable heterogeneity (*I*^2^ = 80%, *p* = 0.002) (Figure 6). There was a very low quality of evidence, according to the GRADE rating.

#### 3.4.4. Physical Performance

Two studies evaluated walking speed at four weeks [35] and 24 weeks [40]. Mori et al. (2022) found no difference between groups [40]. However, Rondanelli et al. (2020) showed statistically significant improvements in walking speed in favor of the RET plus WP supplementation [35]. Three studies evaluated physical performance according to SPPB at four [35] and 12 weeks [36,37]. Amasene et al. (2021) observed no difference between groups [36]. However, Rondanelli et al. (2020) showed statistically significant improvements in physical performance in favor of the RET plus WP group [35]. Only one study evaluated dynamic balance through the TUG test at four weeks [35]. The results indicated that the RET plus WP group was statistically more effective in improving dynamic balance [35].

## 4. Discussion

The aim of this systematic review was to determine the effectiveness of WP supplementation during RET vs. RET with or without placebo supplementation on skeletal muscle mass, muscle strength, and physical performance in older people with Sarcopenia. Our results indicate that WP supplementation associated with RET is effective in increasing skeletal muscle mass according to the appendicular muscle index, and handgrip strength. However, we did not observe differences in appendicular muscle mass between RET plus WP supplementation and RET with or without placebo supplementation. In addition, the increase in handgrip strength did not exceed the minimally important clinical difference [31]. There were insufficient data to perform a meta-analysis on physical performance. The results of the studies were discordant in relation to physical performance.

The effectiveness of adding WP supplementation during RET has been well studied in other older populations [19,20,22,24]. However, systematic reviews focused on older people with Sarcopenia are scarce. In this sense, our results differ partially from the findings of the systematic review by Chang and Choo (2023), whose aim was to evaluate the effectiveness of WP, leucine, and vitamin D supplementation in patients with Sarcopenia [41]. Based on two studies, the meta-analysis by Chang and Choo (2023) showed statistically significant differences in favor of the WP, leucine, and vitamin D group, with moderate effect sizes for skeletal muscle mass and large effect sizes for handgrip strength. The findings of Chang and Choo (2023) are likely to have overestimated effect sizes. Even though our findings are supported by a larger number of studies, the certainty of the evidence demonstrates the need to improve the methodological quality of future studies to obtain more accurate conclusions.

The small increases in skeletal muscle mass and handgrip strength in older people with Sarcopenia could be due to several factors. There are reports demonstrating that older people performing RET (with and without Sarcopenia) present a higher percentage increase in muscle strength than in skeletal muscle mass [42,43]. Additionally, these results could be explained by the characteristics of the sample, the dosage of the interventions, and the scant control of confounding variables, including physical activity and diet intake. Regarding the characteristics of the sample, 69.25% (*n* = 265) of the participants included in our meta-analysis were women. Furthermore, 2/7 of the studies included in this systematic review were conducted only on women [34,37]. This is relevant since it has been observed that older women with Sarcopenia tend to show a lower increase in skeletal muscle mass with RET [44]. On the other hand, the blunted stimulation of muscle protein synthesis rates is known as anabolic resistance, and it has been suggested as a theoretical framework to support interventions in people with Sarcopenia [45]. Aging anabolic resistance is influenced by factors such as digestion, absorption, anabolic signaling proteins, muscle perfusion, splanchnic amino acid sequestration, physical activity levels, and postprandial amino acid availability and delivery [45]. Because of these factors, it has been suggested that a greater amount of WP supplementation (~40 g) [46] together with a RET program could be more effective in increasing skeletal muscle mass. However, the dose of WP ranged from 10 to 22 g in the studies included in the meta-analysis [34,35,36,38,40].

We know that to build skeletal muscle mass, the amount and quality of food consumed (diet) are important. It has been reported that older people with Sarcopenia have alterations in their usual diet intake [47,48,49,50]. However, only three studies controlled diet intake during the intervention period [34,35,40]. In addition, it is well known that levels of physical activity can decrease anabolic resistance, thus increasing the effectiveness of nutritional and exercise interventions [45]. However, only the study by Mori et al. (2022) monitored the levels of physical activity and diet intake before and after the intervention. They found that levels of physical activity and diet intake did not change after the intervention in either group [40]. Their results indicated that there were no differences between RET and RET plus WP supplementation in improving handgrip strength, knee extension strength, and relative skeletal muscle mass [40].

RET programming, to obtain gains in skeletal muscle mass and muscle strength, must be individualized, progressive, and of moderate-high intensity (70–80% of 1RM). However, several studies included in this systematic review performed moderate RET or adapted strength training by performing it with elastic bands or sandbags [35,38,40]. Additionally, training frequency could also influence the effects of RET on skeletal muscle mass. For example, Rondanelli et al. (2016; 2020) conducted training sessions five times a week and found statistically significant differences in appendicular muscle mass and relative skeletal muscle mass in favor of RET plus WP supplementaion. In contrast, studies that had 2–3 weekly sessions found no differences between the groups after the intervention [35,36,38]. Finally, current evidence shows us that evaluating skeletal muscle mass through a Computed Tomography Scan or Magnetic Resonance Imaging (especially changes in the thigh of older people) could be a better way to see changes [51].

Insufficient and controversial scientific evidence exists on the effect of WP supplementation combined with RET on physical performance [35,36,40]. Rondanelli et al. (2020) showed that an intervention with RET plus WP supplementation induces positive effects on walking speed, physical performance using SPPB, and dynamic balance using TUG in older adults with Sarcopenia. This intervention also reduces hospitalization time, which is related to decreased healthcare expenditures and different adverse events associated with hospitalization time [4]. However, the other two studies did not report significant differences in favor of WP supplementation plus RET compared with placebo supplementation plus RET [36,40]. Mori et al. (2022) reported no benefit in both interventions in improving walking speed [40], and Amasene et al. (2021) [36] reported that the physical performance measured using SPPB significantly improved in both intervention groups regardless of protein-enriched supplementation. Nonetheless, in that study [36], the intervention group demonstrated a decrease in the prevalence of frailty, as evidenced by five older adults supplemented with protein who were classified as frail at baseline but were no longer so after the intervention [36]. The results reported by Amasene et al. (2021) [36] emphasize the effectiveness of RET programs alone in improving the physical performance of older adults, as also reported in a recent systematic review [52]. Therefore, the addition of WP supplementation during RET may not be necessary to achieve significant improvements in physical performance among this population, and additional studies are required in this research field to fully understand of the role of WP supplementation on physical performance during RET.

Finally, the role of WP supplementation in the treatment of Sarcopenia has been previously recommended by international consensus [1,27]. Prior research has suggested that physical exercise is beneficial for individuals with Sarcopenia; however, it may not be sufficient alone to achieve significant clinical outcomes. As a result, a combined exercise program along with supplementation of WP, essential amino acids, and vitamin D has shown significant effects when compared with exercise alone [34]. That study highlights the potential benefits of combined interventions to treat Sarcopenia and improve clinical outcomes. Although physical performance is an important clinical measurement to diagnose Sarcopenia and is recommended by international consensus, it was measured only in two studies [34,35]. Therefore, more studies to analyze the effect on all clinical measurements of Sarcopenia (skeletal muscle mass, muscle strength, and physical performance) are necessary.

### Limitations

Our results should be considered with caution due to the following limitations: First, despite searching eight databases, articles in other languages than English, Spanish, or Portuguese could have been excluded. Second, the studies have shown high clinical heterogeneity in terms of WP supplementation and RET dosing. Third, due to the small number of articles included in the quantitative synthesis, the accuracy of the meta-analytic tests used may be affected. Fourth, although it is a consequence of the high clinical heterogeneity of the studies, the quantitative syntheses showed considerable statistical heterogeneity for handgrip strength. Fifth, it was not possible to perform a moderator analysis and assess publication biases due to the limited number of articles included in the meta-analyses.

## 5. Conclusions

RET plus WP supplementation is more effective in increasing skeletal muscle mass and handgrip strength in older people with Sarcopenia compared with RET with or without placebo supplementation. However, the effect sizes were small for skeletal muscle mass, and the handgrip strength did not exceed the minimally important clinical difference. The quality of the evidence was low to very low, according to the GRADE approach. Further studies are needed in this research field. Future research should report in detail the dosage and periodization of RET program, as well as the proportions of other ingredients that are incorporated into WP supplementation. Higher doses of WP supplementation and a higher frequency of intake could probably improve the results obtained to date. Changes in physical activity and diet intake are confounding variables that future studies should control for. Strategies to avoid participant dropout, blinding, and reporting according to clinical trial registration protocols could reduce the risk of bias.

## Figures and Tables

**Table 1:** Characteristics of the studies.

Author and Year	Population Characteristics					Intervention Characteristics					Results		
	Total Sample	Groups		Sarcopenia Diagnosis	Training Level and Comorbidities	Time Intervention and Context	Groups	Supplement		RET	Outcome Measure	Control	Exp
		Control	Exp					Control	Exp				
**Rondanelli et al., 2016** [34]	*N* = 130 ≥65 years	*N* = 61 A = 80.2 ± 8.5 M = 24 W = 37	*N* = 69 A = 80.7 ± 6.2 M = 29 W = 40	DEXA < 7.26 kg/m^2^ for M and <5.5 kg/m^2^ for W.	NT. With comorbidities (NR). Without physical or cognitive impairment.	Twelve weeks. Outpatient in hospital, 5 days/week.	CG = RET + Placebo EG = RET + WP	A total of 32 g isocaloric maltodextrin once a day at 12:00 pm.	A total of 22 g WP enriched with 2.5 g essential Vitamin D and AA once a day at 12:00 pm.	CG and EG = 20 min per day; Strengthen: chair exercise, toe raises, heel, knee. Knee flexion and extension exercises used weights of 0.50 and 1.50 kg. Resistance bands were used for leg extension, knee flexion, and bicep curls. Balance and Gait exercises, tandem stance, and a tandem walk, each type of exercise 8 times, BORG Intensity 12–14.	SMM: RSMM with DEXA (kg/m^2^) MS: Handgrip with hand dynamometer (kg)	SMM (Δ) = −0.06 (0.21, 0.90) MS (Δ) = −0.47 (−1.07, 0, 12)	SMM (Δ) = 0.21 (0.07, 0.35) ^#^ * MS (Δ) = 3.20 (2.23, 4.18) ^#^ *
**Amasene M. et al., 2019** [37]	*N* = 28 >70 years	*N* = 13 A = 81.7 ± 6.45 M = 6 W = 7	*N* = 15 A = 82.9 ± 5.59 M = 8 W = 7	EWGSOP	NT. With comorbidities (NR), without physical or cognitive impairment.	Twelve weeks. Inpatient and outpatient in hospital. Two non-consecutive days/week.	CG = RET + Placebo EG = RET + WP	Placebo with maltodextrin and lemon-flavored hydroxyethylcellulose after each training session.	A total of 20 g WP isolate, enriched with 3 g lemon flavor leucine, after each training session.	CG and EG = 60 min per day. Adapted based on 1RM and then gradually increased the load until reaching 70% of 1RM. Strengthening limbs, 2 set x exercise, load and RM vary by participant; exercises were also practiced to improve dynamic balance.	MS: Handgrip with hand dynamometer (kg/body mass) PP: SPPB total score	MS (post) = 0.3 (0.09) PP (post) = 10.3 (1.89) ^#^	MS (post) = 0.4 (0.09) PP (post) = 11.3 (0.96) ^#^
**Nabuco H. et al., 2019** [39]	*N* = 26 ≥60 years	*N* = 13 A = 70.1 ± 3.9 W = 13	*N* = 13 A = 68.0 ± 4.2 W = 13	DEXA. Assessed body fat mass 35% combined with ALST less than <15.02 kg.	NT. Obesity, HT, DM or HLP. Without physical or cognitive alteration.	Twelve weeks. Outpatient at university, 3 alternate days/week.	CG = RET + Placebo EG = RET + WP	Placebo, after each training session. Maltodextrin only on training days.	A total of 35 g hydrolyzed WP after each training session. Only on training days.	CG and EG = Alternate conventional RET, 3 sets of 8–12 repetitions, loads adjusted individually for each exercise according to their abilities. Exercises: chest press, horizontal leg press, seated row, knee extension, preacher curl (free weights), leg curl, triceps pushdown, and seated calf raise.	MS1: Knee extension (kg). MS2: Chest press (kg) MS3: Preacher curl (kg). PP = 10-m walk test (s)	MS1 (post) = 53.4 ± 9.4 ^#^ MS2 (post) = 42.8 ± 7.1^#^ MS3 (post) = 21.5 ± 2.9 ^#^ PP (post) = 6.8 ± 0.6 ^#^	MS1 (post) = 51.8 ^#^ 10.9 ^#^ MS2 (post) = 44.8 ± 8.6 ^#^ MS3 (post) = 23.7 ± 4.3 ^#^ PP (post) = 6.9 ± 0.8 ^#^
**Rondanelli et al., 2020** [35]	*N* = 127 ≥65 years	*N* = 63 A = 81 ± 5 M = 17 W = 46	*N* = 64 A = 80 ± 7 M = 26 W = 38	EWGSOP 2010.	NT. With OA, COPD, STROKE, fracture, surgery. without physical or cognitive impairment.	Eight weeks. Outpatient in hospital, 5 days/week.	CG = RET + Placebo EG = RET + WP	Isocaloric formula 40 g flavored powder with maltodextrins. Twice a day, once at breakfast and once in the afternoon.	A total of 20 g WP enriched with 2.8 g leucine, 9 g carbohydrates, 3 g fat, 800 IU vitamin D, a mixture of vitamins, 500 mg calcium, and fibers. Twice a day, once at breakfast and once in the afternoon.	CG and EG = RET (Borg 12–14); 20 min, increased by intensity exercises up to 30 min. RET, Strengthening (5–10 min) toe raises, heel raises, knee raises, seated knee extensions, standing hip flexions, and lateral leg raises; weight-bearing ankle exercises with weights ranging from 0.5 to 1.5 kg; Resistance band leg extensions and hip flexions; double arm curls, and bicep curls. Balance, walking (5–10 min), single-leg stands, tandem stands, multi-directional weight shifts, tandem walk.	SMM1: AMM with DEXA (g). SMM2: RSMM with DEXA (kg/m^2^). MS: Handgrip with dynamometer (kg) PP1: SPPB total score PP2: Walk speed with 4 m test (m/s) PP3: TUG	SMM1 (Δ) = −69.4 (−843.7, 704.9) ^#^ SMM2 (Δ) = −0.02 (−0.35, 0.32) MS (Δ) = −1.47 (−2.01, −0.92) ^#^ PP1 (Δ) = 0.33 (0.19, 0.46) ^#^ PP2 (Δ) = 0.06 (0.043, 0.08) ^#^ PP3 (Δ) = −0.76 (−1.07, −0.44)	SMM1 (Δ) = 949.8 (783.7, 1115.8) ^#^ * SMM2 (Δ) = 0.38 (0.31, 0.44) ^#^ * MS (Δ) = 3.98 (3.20, 4.75) ^#^ * PP1 (Δ) = 2.6 (2.23, 2.98) ^#^ * PP2 (Δ) = 0.06 (0.43, 0.08) ^#^ PP3 (Δ) = 2.95 (2.41, 3.49) ^#^ *
**Li Z. et al., 2020** [38]	*N* = 169 ≥60 years	CG1 = 51 A = 70 ± 3 M = 22 W = 29 CG2 = 37 A = 73 ± 5 M = 14 W = 23 CG3 = 48 A = 72 ± 6 M = 12 W = 21	*N* = 59 EG = 33 A = 71.52 ± 5.28 M = 22 W = 37	AWGS 2014.	NT. With DM, HT or HLP.	Twelve weeks. Outpatient in two hospital centers, 3 alternate days/week.	CG1 = WP CG2 = RET CG3 = usual care EG = RET + WP	Without supplementation or placebo.	A total of 10 g WP 3 times/day with food. EPA (300 mg), DHA (200 mg), and vitamin D3 (250 IU) in capsules, with 2 capsules × 2 times a day, 30 min after breakfast and dinner.	CG2 and EG = 30 min + 60 min walk GE = Strengthening (20 min) and slow walking (5 min) 8RM focused on limbs using dumbbells and sandbags. Outdoor activity refers to a one-hour walk with sun exposure 3 days/week on the days opposite resistance training. The speed should be more than 800 steps in 10 min.	SMM1: AMM with M-BIA (kg). SMM2: RSMM with M-BIA (kg/m^2^). MS: Handgrip with hand dynamometer (kg)	SMM1 (post) = 15.00 ± 3.00 SMM2 (post) = 6.09 ± 0.73 HS (post) = 23.62 ± 5.83	SMM1(post) = 16.21 ± 3.59 * SMM2 (post) = 6.32 ± 0.84 * HS (post) = 24.83 ± 6.26 *
**Amasene et al., 2021** [37]	*N* = 41 ≥70 years	*N* = 20 A = 81.2 ± 6.14 W = 20	*N* = 21 A = 82.9 ± 5.67 W = 21	EWGSOP 2018.	NT. With comorbidities, without physical or cognitive impairment.	Twelve weeks. Inpatient and outpatient in hospital. Two non-consecutive.	CG = RET. EG = RET + WP	Placebo with maltodextrin and lemon-flavored hydroxyethylcellulose after each training session.	A total of 20 g isolate WP enriched with 3 g leucine once daily post-exercise.	CG and EG = Supervised training, 60 min per day. Adapted based on 1RM and then gradually increased the load until reaching 70% of 1RM.	SMM1: AMM with DEXA (kg). SMM2: RSMM with DEXA (kg/m^2^). MS: Handgrip with hand dynamometer (kg) PP: SPPB total score	SMM1 (post) = 18.5 ± 3.6 SMM2 (post) = 7.5 ± 1.16 MS (post) = 24.5 ± 7.32 PP (post) = 10.3 ± 1.89 ^#^	SMM1 (post) = 17.3 ± 2.78 SMM2 (post) = 6.9 ± 0.66 MS (post) = 26.6 ± 6.50 PP (post) = 11.3 ± 0.96 ^#^
**Mori et al., 2022** [40]	*N* = 70 ≥65 years	CG1 = 23 A = 77.6 ± 5.2 M = 4 W = 19 CG2 = 24 A = 77.8 ± 4.5 M = 16 W = 8	EG = 23 A = 77.7 ± 3.3 M = 3 W = 20	AWGS 2014.	NT. With comorbidities (NR), without physical or cognitive impairment.	Twenty-four weeks. Outpatient in hospital, 2 days/week.	CG1 = RET CG2 = WP EG = RET + WP	Without supplementation or placebo.	A total of 11 g of protein, which contains 160 kcal of energy, 2.2 g of fat, 24 g of carbohydrates, and 2300 mg of leucine per serving. It was used after 3 h of lunch.	CG1 and EG = 30–40 min. Elastic resistance band exercises, resistance exercises with body weight load 50–70% of 1RM, 2–3 sets.	SMM: RSMM with M-BIA (kg/m^2^). MS1: Handgrip with hand dynamometer (kg) MS2: Knee extension with hand-held dynamometer (kg) PP: Usual walking speed (m/s)	SMM (post) = 5.39 ± 0.92 ^#^ MS1 (post) = 16.8 ± 3.0 MS2 (post) = 14.6 ± 5.9 ^#^ PP (post) = 1.03 ± 0.27	SMM (post) = 5.51 ± 0.66 ^#^ MS1 (post) = 17.6 ± 3.4 ^#^ MS2 (post) = 14.6 ± 3.0 ^#^ PP (post) = 1.03 ± 0.24

**Abbreviations:** 1RM: one-repetition maximum; A: age; AA: amino acid; ALST: arms and legs soft tissue; AMM: appendicular muscle mass; AWGS: Asian Working Group for Sarcopenia; CG: control group; COPD: chronic obstructive pulmonary disease; DHA: docosahexaenoic acid; DM: diabetes mellitus; DEXA: Dual-Energy X-ray Absorptiometry; EG: experimental group; EPA: Eicosapentaenoic acid; EWGSOP: European Working Group on Sarcopenia in Elderly People; HLP: hyperlipidemia; HT: hypertension; M: men; M-BIA: multi-frequency bioelectrical impedance analysis; MS: muscle strength; N: number of participants; NR: not reported; NT: not trained; OA: osteoarthritis; PP: physical performance; RET: resistance exercise training; RSMM: relative skeletal muscle mass; SMM: skeletal muscle mass; SPPB: short physical performance battery; W: women; WP: whey protein. * Statistically significant differences between groups *p* < 0.05. ^#^ Statistically significant differences intragroup *p* < 0.05. Δ difference pre-post intervention.

## Data Availability

Data are contained within the article.

## Supplementary Materials

The following supporting information can be downloaded at: https://www.mdpi.com/article/10.3390/nu15153424/s1, Table S1: Search strategies; Table S2: Excluded studies; Table S3: Grading of Recommendation, Assessment, Development, and Evaluation (GRADE); Table S4: Weekly improvements between RET plus WP and RET with or without placebo.
