# Supplementary material for: Effectiveness of Whey Protein Supplementation during Resistance Exercise Training on Skeletal Muscle Mass and Strength in Older People with Sarcopenia: A Systematic Review and Meta-Analysis
Source: Nutrients. 2023 Aug 2;15(15):3424. doi: 10.3390/nu15153424 (PMC10421506; doi:10.3390/nu15153424)
Supplement: Supplementary file 1 [file nutrients-15-03424-s001.zip › nutrients-2446503-supplementary.pdf]

Supplementary material

**Table S1.** Search strategies

| Database | Search strategy | Results |
|---|---|---|
| Pubmed | ((((((((sarcopenia) OR (Sarcopenic)) OR (sarcopaenia)) OR (Sarcopen))) AND ((((((Strength training) OR (Strength exercise)) OR (Weight lifting)) OR (Resistance training)) OR (Resistance exercise)))) AND ((((((Whey protein) OR (Protein supplement)) OR (whey supplement)) OR (Whey intake)) OR (Protein intake)))) AND ((((Controlled Clinical Trial) OR (randomized controlled trial)) OR (Clinical trial)))) | 157 |
| EMBASE | ('sarcopenia'/exp OR sarcopenia OR sarcopenic OR sarcopaenia OR sarcopen) AND ('strength training'/exp OR 'strength training' OR (('strength'/exp OR strength) AND ('training'/exp OR training)) OR 'strength exercise'/exp OR 'strength exercise' OR (('strength'/exp OR strength) AND ('exercise'/exp OR exercise)) OR 'weight lifting'/exp OR 'weight lifting' OR (('weight'/exp OR weight) AND ('lifting'/exp OR lifting)) OR 'resistance training'/exp OR 'resistance training' OR (('resistance'/exp OR resistance) AND ('training'/exp OR training)) OR 'resistance exercise'/exp OR 'resistance exercise' OR (('resistance'/exp OR resistance) AND ('exercise'/exp OR exercise))) AND ('whey protein'/exp OR 'whey protein' OR (('whey'/exp OR whey) AND ('protein'/exp OR protein)) OR 'protein supplement' OR (('protein'/exp OR protein) AND supplement) OR 'whey supplement' OR (('whey'/exp OR whey) AND supplement) OR 'whey intake' OR (('whey'/exp OR whey) AND intake) OR 'protein intake'/exp OR 'protein intake' OR (('protein'/exp OR protein) AND intake)) AND ('controlled clinical trial'/exp OR 'controlled clinical trial' OR (controlled AND ('clinical'/exp OR clinical) AND ('trial'/exp OR trial)) OR 'randomized controlled trial'/exp OR 'randomized controlled trial' OR (randomized AND controlled AND ('trial'/exp OR trial)) OR 'clinical trial'/exp OR 'clinical trial' OR (('clinical'/exp OR clinical) AND ('trial'/exp OR trial))) | 270 |
| SCOPUS | TITLE-ABS-KEY ( ( ( ( ( ( ( ( sarcopenia ) OR ( sarcopenic ) ) OR ( sarcopaenia ) ) OR ( sarcopen ) ) ) AND ( ( ( ( ( ( strength AND training ) OR ( strength AND exercise ) ) OR ( weight AND lifting ) ) OR ( resistance AND training ) ) OR ( resistance AND exercise ) ) ) ) AND ( ( ( ( ( ( whey AND protein ) OR ( protein AND supplement ) ) OR ( whey AND supplement ) ) OR ( whey AND intake ) ) OR ( protein AND intake ) ) ) ) AND ( ( ( ( controlled AND clinical AND trial ) OR ( randomized AND controlled AND trial ) ) OR ( clinical AND trial ) ) ) ) ) | 293 |
| LILACS | ((((((((sarcopenia) OR (Sarcopenic)) OR (sarcopaenia)) OR (Sarcopen))) AND ((((((Strength training) OR (Strength exercise)) OR (Weight lifting)) OR (Resistance training)) OR (Resistance exercise)))) AND ((((((Whey protein) OR (Protein supplement)) OR (whey supplement)) OR (Whey intake)) OR (Protein intake)))) AND ((((Controlled Clinical Trial) OR (randomized controlled trial)) OR (Clinical trial)))) | 0 |
| CINHAL | ((((((((sarcopenia) OR (Sarcopenic)) OR (sarcopaenia)) OR (Sarcopen))) AND ((((((Strength training) OR (Strength exercise)) OR (Weight lifting)) OR (Resistance training)) OR (Resistance exercise)))) AND ((((((Whey protein) OR (Protein supplement)) OR (whey supplement)) OR (Whey intake)) OR (Protein intake)))) AND ((((Controlled Clinical Trial) OR (randomized controlled trial)) OR (Clinical trial)))) | 33 |
| SPORTDISCUS | ((((((((sarcopenia) OR (Sarcopenic)) OR (sarcopaenia)) OR (Sarcopen))) AND ((((((Strength training) OR (Strength exer- | 11 |

| | | |
|---|---|---|
| | cise)) OR (Weight lifting)) OR (Resistance training)) OR (Resistance exercise)))) AND ((((((Whey protein) OR (Protein supplement)) OR (whey supplement)) OR (Whey intake)) OR (Protein intake)))) AND ((((Controlled Clinical Trial) OR (randomized controlled trial)) OR (Clinical trial)))) | |
| Web of science | ((((((((sarcopenia) OR (Sarcopenic)) OR (sarcopenia)) OR (sarcoped))) AND ((((((Strength training) OR (Strength exercise)) OR (Weight lifting)) OR (Resistance training)) OR (Resistance exercise)))) AND ((((((Whey protein) OR (Protein supplement)) OR (whey supplement)) OR (Whey intake)) OR (Protein intake)))) AND ((((Controlled Clinical Trial) OR (randomized controlled trial)) OR (Clinical trial)))) (All Fields) | 223 |
| Epistemonikos | ((((((((sarcopenia) OR (Sarcopenic)) OR (sarcopaenia)) OR (Sarcopen))) AND ((((((Strength training) OR (Strength exercise)) OR (Weight lifting)) OR (Resistance training)) OR (Resistance exercise)))) AND ((((((Whey protein) OR (Protein supplement)) OR (whey supplement)) OR (Whey intake)) OR (Protein intake)))) AND ((((Controlled Clinical Trial) OR (randomized controlled trial)) OR (Clinical trial)))) | 60 |
| | Total | 1047 |

* All searches were carried out on january 24, 2023.

**Table S2.** Excluded studies.

| | Reference | Reason |
|---|---|---|
| 1 | Björkman, Mikko P., Merja H. Suominen, Hannu Kautiainen, Satu K. Jyväkorpi, Harriet U. Finne-Soveri, Timo E. Strandberg, Kaisu H. Pitkälä, y Reijo S. Tilvis. «Effect of Protein Supplementation on Physical Performance in Older People With Sarcopenia–A Randomized Controlled Trial». Journal of the American Medical Directors Association 21, n.º 2 (february 2020): 226-232.e1. https://doi.org/10.1016/j.jamda.2019.09.006. | No resistance exercise |
| 2 | Dimori, Sergio, Giorgio Leoni, Luca Fior, y Fulvio Gasparotto. «Clinical Nutrition and Physical Rehabilitation in a Long-Term Care Setting: Preliminary Observations in Sarcopenic Older Patients». Aging Clinical and Experimental Research 30, n.º 8 (August 2018): 951-58. https://doi.org/10.1007/s40520-017-0859-8. | Observational study |
| 3 | Kemmler, Wolfgang, Matthias Kohl, Franz Jakob, Klaus Engelke, y Simon von Stengel. «Effects of High Intensity Dynamic Resistance Exercise and Whey Protein Supplements on Osteosarcopenia in Older Men with Low Bone and Muscle Mass. Final Results of the Randomized Controlled FrOST Study». Nutrients 12, n.º 8 (August 2020): 2341. https://doi.org/10.3390/nu12082341. | Control group without resistance exercise |
| 4 | Kim, Hunkyung, Miji Kim, Narumi Kojima, Ken Fujino, Erika Hosoi, Hisamine Kobayashi, Shinji Somekawa, Yoshifumi Niki, Yukari Yamashiro, y Hideyo Yoshida. «Exercise and Nutritional Supplementation on Community-Dwelling Elderly Japanese Women With Sarcopenic Obesity: A Randomized Controlled Trial». Journal of the American Medical Directors Association 17, n.º 11 (November 2016): 1011-19. https://doi.org/10.1016/j.jamda.2016.06.016. | Supplement not based on whey protein |
| 5 | Liao, Chun-De, Yi-Hung Liao, Tsan-Hon Liou, Ching-Ya Hsieh, Yu-Chi Kuo, y Hung-Chou Chen. «Effects of Protein-Rich Nutritional Composition Supplementation on Sarcopenia Indices and Physical Activity during Resistance Exercise Training in Older Women with Knee Osteoarthritis». Nutrients 13, n.º 8 (July 2021): 2487. https://doi.org/10.3390/nu13082487. | Population with and without sarcopenia |
| 6 | Maltais, Mathieu L., Joëlle P. Ladouceur, y Isabelle J. Dionne. «The Effect of Resistance Training and Different Sources of Postexercise Protein Supplementation on Muscle Mass and Physical Capacity in Sarcopenic Elderly Men». Journal of Strength and Conditioning Research 30, n.º 6 (June de 2016): 1680-87. https://doi.org/10.1519/JSC.0000000000001255. | Supplement not based on whey protein |
| 9 | Mori, Hiroyasu, y Yasunobu Tokuda. «Effect of Whey Protein Supplementation after Resistance Exercise on the Muscle Mass and Physical Function of Healthy Older Women: A Randomized Controlled Trial: Nutritional Supplementation for Sarcopenia». Geriatrics & Gerontology International 18, n.º 9 (September 2018): 1398-1404. https://doi.org/10.1111/ggi.13499. | Healthy older women |
| 10 | Rondanelli, M., E. Cereda, C. Klersy, M.A. Faliva, G. Peroni, M. Nichetti, C. Gasparri, D. Spadaccini, V. Infantino, y S. Perna. «CN06: Improving Rehabilitation in Sarcopenia (IRIS) by Muscle-Targeted Nutritional Support: A Randomized, Double-Blind, Placebo-Controlled Trial». Clinical Nutrition 38 (September 2019): S3. https://doi.org/10.1016/S0261-5614(19)32121-1. | Congress article |
| 11 | Seo, Myong-Won, Sung-Woo Jung, Sung-Woo Kim, Jung-Min Lee, Hyun Chul Jung, y Jong-Kook Song. «Effects of 16 Weeks of Resistance Training on Muscle Quality and Muscle Growth Factors in Older Adult Women with Sarcopenia: A Randomized Controlled Trial». International Journal of Environmental Research and Public Health 18, n.º 13 (June 2021): 6762. https://doi.org/10.3390/ijerph18136762. | Supplement not based on whey protein |

| | | |
|---|---|---|
| 12 | Yoshimura, Yoshihiro, K. Uchida, S. Jeong, y M. Yamaga. «Effects of Nutritional Supplements on Muscle Mass and Activities of Daily Living in Elderly Rehabilitation Patients with Decreased Muscle Mass: A Randomized Controlled Trial». The Journal of Nutrition, Health & Aging 20, n.º 2 (February 2016): 185-91. https://doi.org/10.1007/s12603-015-0570-4. | Supplement not based on whey protein |
| 13 | Zhao, Jiaxi, Yiqin Huang, y Xiaofeng Yu. «Effects of Nutritional Supplement and Resistance Training for Sarcopenia in Patients with Inflammatory Bowel Disease: A Randomized Controlled Trial». Medicine 101, n.º 34 (August 2022): e30386. https://doi.org/10.1097/MD.0000000000030386. | Does not specify exercise intensity |

**Table S3.** Grading of Recommendation, Assessment, Development and Evaluation (GRADE).

| Certainty assessment | | | | | | | № of patients | | Effect | | Certainty | Importance |
|---|---|---|---|---|---|---|---|---|---|---|---|---|
| № of studies | Study design | Risk of bias | Inconsistency | Indirectness | Imprecision | Other considera-tions | Exercise plus Whey protein | Exercise | Relative (95% CI) | Absolute (95% CI) | | |
| **Muscle mass: Appendicular muscle index 4-24 weeks** | | | | | | | | | | | | |
| 5 | randomised trials | very serious | not serious | not serious | not serious | none | 230 | 222 | - | SMD **0.24 SD more** (0.05 more to 0.42 more) | ⨁⨁◯◯ Low | NOT IMPORTANT |
| **Muscle mass: Appendicular muscle index (4-12 weeks)** | | | | | | | | | | | | |
| 4 | randomised trials | very serious | not serious | not serious | not serious | none | 207 | 199 | - | SMD **0.26 SD more** (0.05 more to 0.47 more) | ⨁⨁◯◯ Low | NOT IMPORTANT |
| **Muscle mass: Appendicular muscle mass (4-12 weeks)** | | | | | | | | | | | | |
| 3 | randomised trials | very serious | not serious | not serious | not serious | none | 138 | 138 | - | SMD **0.15 SD more** (-0.08 fewer to 0.39 more) | ⨁⨁◯◯ Low | NOT IMPORTANT |
| **Muscular strength: Handgrip (4-24 weeks)** | | | | | | | | | | | | |
| 5 | randomised trials | very serious | serious | not serious | not serious | none | 230 | 222 | - | MD **2.31 Kg more** (0.01 more to 4.60 more) | ⨁◯◯◯ Very low | IMPORTANT |
| **Muscular strength: Handgrip (4-12 weeks)** | | | | | | | | | | | | |
| 4 | randomised trials | very serious | serious | not serious | not serious | none | 207 | 199 | - | MD 2.71 **Kg more** (0.06 more to 5.36 more) | ⨁◯◯◯ Very low | IMPORTANT |

**Abbreviations: CI:** confidence interval; **MD:** mean difference; **SMD:** standardised mean difference

**Table S4.** Weekly improvements between RET plus WP and RET with or without placebo.

| Outcome | Study | SMD | MD (KG) | Training duration (weeks) | Weekly improvement |
|---|---|---|---|---|---|
| **Appendicular muscle index** | Amasene et al., 2021 | -0.09 | - | 12 | -0.01 |
| | Li et al., 2020 | 0.13 | - | 12 | 0.01 |
| | Rondanelli et al, 2020 | 0.52 | - | 4 | 0.13 |
| | Rondanelli et al., 2016 | 0.21 | - | 12 | 0.02 |
| | Mori & Tokuda., 2018 | 0.04 | - | 24 | 0.00 |
| **Appendicular muscle mass** | Amasene et al., 2021 | -0.05 | - | 12 | 0.00 |
| | Li et al., 2020 | 0.07 | - | 12 | 0.01 |
| | Rondanelli et al, 2020 | 0.27 | - | 4 | 0.07 |
| **Handgrip Strength** | Amasene et al., 2021 | - | -0.6 | 12 | -0.05 |
| | Li et al., 2020 | - | 0.28 | 12 | 0.02 |
| | Rondanelli et al, 2020 | - | 5.45 | 4 | 1.36 |
| | Rondanelli et al., 2016 | - | 3.67 | 12 | 0.31 |
| | Mori et al, 2022 | - | 0.9 | 24 | 0.04 |

SMD: standardised mean difference
